# Effect of Functional Progressive Resistance Exercise on Lower Extremity Structure, Muscle Tone, Dynamic Balance and Functional Ability in Children with Spastic Cerebral Palsy

**DOI:** 10.3390/children7080085

**Published:** 2020-07-31

**Authors:** Hye-Jin Cho, Byoung-Hee Lee

**Affiliations:** 1 Graduate School of Physical Therapy, Sahmyook University, Seoul 01795, Korea; manje50@hanmail.net; 2 Department of Physical Therapy, Sahmyook University, Seoul 01795, Korea

**Keywords:** cerebral palsy, functional progressive resistance exercise, muscle strength, muscle tone

## Abstract

The purpose of this study was to investigate the effects of functional progressive resistance exercise (FPRE) on muscle tone, dynamic balance and functional ability in children with spastic cerebral palsy. Twenty-five subjects were randomized into two groups: the FPRE group (*n* = 13) and the control group (*n* = 12). The experimental group participated in an FPRE program for 30 min per day, three times per week for six weeks. Knee extensor strength, rehabilitative ultrasound imaging (RUSI), muscle tone, dynamic balance, and functional ability was evaluated. The results showed statistically significant time × group interaction effects on the dominant side for knee extensor strength and cross-sectional area (CSA) in RUSI (*p* < 0.05). On both sides for thickness of the quadriceps (TQ) in RUSI, muscle tone and dynamic balance were statistically significant time × group interaction effects (*p* < 0.05). Additionally, knee extensor strength, CSA, TQ in RUS, muscle tone, dynamic balance and gross motor function measure (GMFM) in functional ability were significantly increased between pre- and post-intervention within the FPRE group (*p* < 0.05). The results suggest that FPRE is both feasible and beneficial for improving muscle tone, dynamic balance and functional ability in children with spastic cerebral palsy.

## 1. Introduction

Cerebral palsy has been defined as a nonprogressive disorder that affects the development of movement and posture, causing limitation of activity in the developing fetus or infant. Disturbances of sensation, cognition, communication, perception and behavior by seizure disorder are the most common disorders associated with cerebral palsy (CP) [1]. CP can be classified based on the type of movement disorder as spastic, athetoid, ataxic and mixed; CP can also be classified based on the area of the body involved as hemiplegia, diplegia and quadriplegia [2], in which spastic diplegia is the most common type [3]. Spasticity, caused by damage to the pyramidal parts of the brain, is defined as a velocity-dependent resistance to stretch [2]. Due to spasticity, the onset of postural muscle activity in children with CP is delayed compared to normally developing children. In addition, impairment was observed upon sequencing of multiple muscle; additionally, there is an increased level of co-activation of agonist and antagonist muscles at a joint, which results in reduction of balance [4].

CP is a neurological disorder that can cause secondary changes in the musculoskeletal system, such as decreased muscle strength, tightness or contractures around joints and abnormalities in both bony structures and gait [5]. Therefore, children with CP show weakened muscle due to lack of motor unit activation and thickness in 50% of small muscles, compared to normally developing children. Infants with CP have reduced knee extensor and ankle plantar flexor strength than normally developing infants [6], and CP has reduced rectus femoris thickness compared with normally developing children [7]. The thickness of the quadriceps muscle, indicative of lower extremity strength, has an effect on the quality of life of children with CP; indeed, a study has shown that children with thicker quadricep muscles participated more in community-related activities [8]. Based on this result, lower extremity strengthening should be emphasized in the rehabilitation of children with CP.

Many methods of muscle strengthening are recommended for children with CP, such as functional progressive resistance exercise (FPRE) [9], isokinetic training [10], bicycle and treadmill exercise [11], weight training [12], aquatic training [13], sports and recreation [14] and electrotherapy [15]. Until recently, strength training in children with CP was considered inappropriate, as it was believed to lead to increased spasticity or abnormal movement patterns.

The muscles of children with CP have an increased amount of collagen, which hinders movement. This increase in collagen is responsible for contracture development, thereby affecting the passive viscoelastic features of muscle and exerting an impact on the internal resistance of muscle when passive movement of the joint is performed [16]. A weak agonist muscle may not allow full lengthening of the spastic antagonist muscle, leading to contracture development, and an increase in passive tension leads to muscle weakness [17].

FPRE can improve lower limb muscle strength and improve function in children with CP without increasing spasticity [18]. Essentially, FPRE provides sufficient resistance so that a low number of repetitions (usually 8–12) can be completed before fatigue sets in [19]. FPRE includes exercises, such as sit to stand, half-kneeling standing and side step-up [20]. A study on antigravity close kinematic chain exercise [21] showed that FPRE effectively increases lower muscle strength, thereby facilitating lower extremity co-contraction and allowing agonist and antagonist muscles to work effectively; this leads to reduction of muscle tone in the lower extremity [22].

This study aims to contribute to the improvement of rehabilitation in children with spastic CP by investigating the effect of FPRE on knee extensor strength, myoarchitectonic of the quadriceps, muscle tone, dynamic balance and functional ability of the lower extremity.

## 2. Materials and Methods

### 2.1. Methods

The subjects were selected from 28 children with diplegia CP undergoing physical therapy at K Hospital in Gyeonggi-do, Korea. The specific selection criteria of the study subjects were children between the ages of 6 and 13 years diagnosed with diplegic CP, who were able to follow the researcher’s instructions and had a GMFCS (gross motor function classification system) level between I and III [23]. Children were excluded if they had unstable seizures, had received treatment for spasticity or any surgical procedure up to 3 months (for botulinum toxin type A injections) to 6 months (for surgery) prior to the start of the study—or if they suffered from other diseases that interfered with physical activity [24].

Subject’s age, height, weight, BMI and GMFCS level were measured prior to each intervention to apply the appropriate amount of weight for each intervention. All subjects picked a black or white stone from a box containing 28 stones. Subjects were randomly divided into an experimental group or a control group, with 14 subjects in each group.

One week before training and one week after training proceeded the evaluation. The intervention group performed FPRE for 30 min per day, three times per week, during a period of 6 weeks. For the control group, a conventional physical therapy program was applied instead of FPRE. However, during the intervention, one subject in the FPRE group had to drop out due to their health condition and two subjects in the control group were excluded because they moved out of town.

This study was conducted with the approval of the research institutional review board of Sahmyook University (2-7001793-AB-N-012018014HR) and it was registered (KCT0005055) as a Clinical Research Information Service (CRIS) in Republic of Korea. The objective and the procedures performed in the study were explained to the subjects’ parents, and all of the subjects’ parents provided informed consent for inclusion in the study. Therefore, this study was conducted according to the ethical principles of the Declaration of Helsinki.

### 2.2. Experimental Methods

#### 2.2.1. Functional Progressive Resistance Exercise

The FPRE program was modified based on circuit training that follows the program used by US National strength and conditioning association (NSCA). Strength training must be individualized and should involve a progressive increase in intensity to be successful, thereby stimulating strength gains that are greater than those associated with normal growth and development [25]. The FPRE can be used to bear, overcome or resist force, such as body weight, free weights or machines. The exercise was conducted three times per week for 6 weeks. Each exercise was comprised of 5 min of warmup exercise followed by three different types of exercise. Exercise repetition increased to five times in the first two weeks, 10 times in the subsequent 2 weeks and 15 times in the last two weeks. More specifically, according to subject’s participation, body weight and exercise repetition will be increased every two weeks by 5%, 10% and 35% based on their body weight. According to each subject’s performance both weight and repetition would be increased; however, in the event that the subjects were unable to follow the increase in exercise repetition or weights used during exercise, the level of difficulty would remain the same.

In the following protocol, three circuit exercises were included: sit to stand, half-kneeling standing up and side step-up. In the sit to stand exercise, the child sits on a bench with no back rest. In the starting position, the child’s back, knee and ankle need to be flexed at a 90° angle and their ankles should be in contact with the floor. From the starting position, the subject would be instructed by the physical therapist to stand up slowly from the bench. In the half-kneeling standing exercise, the child is sitting in a half-kneeling position without any external support. From this starting position, the child gradually pushes forward to stand up while the weight is shifted forward on the front leg. In the side step-up exercise, the child climbs up a 15 cm staircase sideways [26]. Between each circuit, 30 s to 1 min of rest time was given to subjects. Longer rest times were given to subjects with lower GMFCS scores to reduce stress. A cooling down exercise and range of motion stretching was held in the final 2 min (Table 1).

#### 2.2.2. Conventional Therapy

Conventional therapy, which was prescribed by a rehabilitation doctor in K hospital, included FES, standing frame and mat exercise. In the control group, conventional therapy had a duration of 30 min three times per a week for 6 weeks. The instructor for each exercise was a pediatric physiotherapist with 3 or more years of work experience.

### 2.3. Outcome Measurements

#### 2.3.1. Knee Extensor Strength

In this study, knee extensor strength was measured with a handheld dynamometer FPX 50 (Wagner, Inc., Greenwich, CT, USA, 2017) before and after the intervention by therapists who received 40 min of education regarding proper use of the hand hold dynamometer. The measurement of the knee extensor was performed with the subject in a sitting position, with knee and hip in a 90-degree flexion without back support. Since gravity effects can result in measurement errors, all actions were tested in gravity-neutralized Bryant positions [27]. Subjects were required to place both hands on their lap and HHD was placed 3 cm above the ankle joint. Three attempts were made to find the mean value for the knee extensor strength measurement. The reliability ICC was 0.91 [28].

#### 2.3.2. Rehabilitative Ultrasound Imaging

The use of ultrasound imaging (USI) to aid rehabilitation of neuromusculoskeletal disorders or rehabilitative ultrasound imaging (RUSI), is defined as ‘a procedure used by physical therapists to evaluate muscle and related soft tissue morphology and function during exercise and physical tasks [29]. In this study, morphology of the quadriceps muscle was measured with portable ultrasound, Medison Mysono *P*-US system (U5, Samsung Medison, Seoul, Korea). The cross-sectional area of the rectus femoris and the thickness of the quadriceps, from the top of the rectus femoris to the bottom of the vastus intermedius, were measured three times on both legs. Regarding the reliability of this test, the interrater reliability ICC was 0.87–0.97, while the intra-rater reliability ICC was 0.78–0.95 in younger people [30].

#### 2.3.3. Muscle Tone

In this study, Electronic goniometer, Baseline 12-1027 Absolute+Axis digital goniometer (Baseline, Inc., New York, NY, USA, 2016) was used to measure the popliteal range of motion in passive, speed and active. In supine position ipsilateral hip and knee were flexed to 90° and the knee maximally passively extended to the point of mild resistance, active range of motion and range of motion with velocity were also measured in same positions [31]. To provide consistent rate and provide highly reliable measures, it was calculated as the mean of three trials. The ICC for this test was 0.999 [32].

#### 2.3.4. Dynamic Balance

In this study, dynamic balance was examined using the functional reach test (FRT). The FRT was performed with a leveled yardstick that was mounted on the wall at the height of the patient’s acromion level in the unaffected arm while sitting in a chair. Hips, knees and ankles were positioned at a 90-degree flexion, with feet positioned flat on the floor. The initial reach is measured with the patient sitting against the back of the chair with the upper extremity flexed to 90 degrees; the measurement was made from the distal end of the third metacarpal along the yardstick [33]. The FRT measures the maximum distance that subjects can reach forward (F-FRT) and sideways (S-FRT) with their arm while maintaining a fixed base of support in the sitting position. The distance was measured in centimeters to the second digit. The interrater reliability ICC of this test was 0.99 and intra-rater reliability ICC was 0.97 [34].

#### 2.3.5. Functional Ability

Functional ability was scored with the GMFM-88. The gross motor function measure (GMFM) is a five-level classification system that appears to be valid in assessing the child’s current motor functions, including laying/rolling, sitting, crawling/kneeling, standing and walking/running/jumping and is thought to have prognostic potential, i.e., early classification of a child could help determine long-term motor function [35]. The reliability ICC ranged from 0.92 to 0.99 for all dimensions and total scores [36].

### 2.4. Statistical Analysis

All demographic variables of subjects displayed normal distribution. SPSS version 25.0 statistical software (IBM, Chicago, IL, USA) was used for analysis of all statistical values. Results are presented as mean ± standard deviation. The general characteristics of two groups were analyzed using chi-squared analysis and the independent *t*-test. The interaction effect between group and time was assessed using a repeated-measures analysis of variance. A paired *t*-test was used to compare the results before and after the intervention in each FPRE group and control group. For all tests, the level of statistical significance was set to 0.05.

## 3. Results

### 3.1. General Characteristics of Subject

Demographic characteristics are shown in Table 2. No significant differences were observed in the baseline value between the FPRE group and control group for all parameters.

### 3.2. Comparison of Knee Extensor Muscle Strength between the FPRE Group and Control Group

Statistically significant time factor effects on knee extensor muscle strength of the dominant and non-dominant side (*p* < 0.05) were observed, as well statistically significant time × group interaction effects on the knee extensor muscle strength of the dominant side (*p* < 0.05).

A paired *t*-test revealed statistically significant improvements on the knee extensor muscle strength of the dominant and non-dominant side in the FPRE group (*p* < 0.05). However, in the control group, the mean value between the pre and posttest showed no significant difference (Table 3).

### 3.3. Comparison of the Structure of the Quadriceps between the FPRE Group and the Control Group

Changes in the lower extremity, specifically the quadriceps, were assessed with portable RUSI. Table 4 presents the results observed in the FPRE group and the control group. Statistically significant time factor effects on the mean value of TQ and CSA of the dominant and non-dominant side were observed (*p* < 0.05), as well as statistically significant group factor effects on the CSA of the dominant and non-dominant side. Additionally, statistically significant time × group interaction effects were observed on the mean value of TQ of the dominant and non-dominant side and CSA of the dominant side (*p* < 0.05).

A paired *t*-test revealed a statistically significant increase on the mean value of TQ and CSA of the dominant and non-dominant sides in the FPRE group after the intervention (*p* < 0.05). However, the mean value of TQ of the dominant side significantly increased after the intervention in the control group (*p* < 0.05).

### 3.4. Comparison of Muscle Tone According to Popliteal Angle in Passive, Speed and Active Ranges of Motion between FPRE and Control Group

Popliteal angles in passive, speed and active ranges of motion were assessed to evaluate the effects of FPRE on lower leg range of motion and strength. Statistically significant time factor effects on the PA-P of the dominant side and PA-A of the dominant and non-dominant side were observed (*p* < 0.05). Statistically significant group factor effects were observed on the PA-P of the dominant and non-dominant side, as well as PA-S of the non-dominant side (*p* < 0.05). In addition, statistically significant time × group interaction effects were observed on the PA-P, PA-S and PA-A of the dominant and non-dominant sides (*p* < 0.05).

A paired *t*-test revealed a statistically significant increase after the intervention on the PA-P and PA-A of the dominant and non-dominant sides and PA-S of the non-dominant side in the FPRE group (*p* < 0.05). However, in the control group, the mean value between the pre and posttest showed no significant difference (Table 5).

### 3.5. Comparison of Dynamic Balance between the FPRE Group and the Control Group

Dynamic balance was assessed with the modified FRT in two different positions: forward reaching position and side reaching position. Statistically significant time factor effects were observed on the S-FRT (*p* < 0.05) and statistically significant time × group interaction effects were observed on the forward functional reach test (F-FRT) and side functional reach test (S-FRT) (*p* < 0.05).

A paired *t*-test revealed a statistically significant increase on the F-FRT and S-FRT in the FPRE group after the intervention (*p* < 0.05). However, in the control group, the mean value between the pre and posttest showed no significant difference (Table 6).

### 3.6. Comparisons of the GMFM Score between the FPRE Group and the Control Group

Functional ability was assessed with the GMFM-88. A paired *t*-test revealed a statistically significant increase on the GMFM score in the FPRE group after the intervention (*p* < 0.05). However, in the control group, the mean value between the pre and posttest showed no significant difference (Table 7).

## 4. Discussion

### 4.1. Knee Extensor Muscle Strength

CP affects physical activity and has a negative impact on the child’s physical development. The spasticity and loss of strength experienced by children with CP results increased incidence of gait disorders and increased energy consumption in comparison with their healthy peers [9,37]. The muscle weakness in the lower extremity is particularly important for ambulation and requires strength training in children with CP [38]. Several studies have provided adequate evidence of a correlation between muscle strength and lower extremity function [39,40]. Indeed, increase in lower extremity muscle strength leads to positive effects on functional activities and flexibility [41].

In this study, FPRE strength training programs were used. Using the handheld dynamometer to examine the knee extensor strength, time factor effects were observed on the knee extensor muscle strength of the dominant side and non-dominant side (*p* < 0.05) and time × group interaction effects were observed on the knee extensor muscle strength of the dominant side (*p* < 0.05); these results are consistent with the results of previous studies, suggesting that strength training in CP leads to increased lower extremity strength [24]. The protocol for increasing muscle strength of the knee extensor in CP are numerous; however, due to the low methodological quality of previous studies, the effects of the study protocols may have been overestimated [42]. Nevertheless, we believe that organized method, resistance and repetitions of the exercises can increase lower extremity strength. Anttila et al. [43] reported that strengthen training in children with CP is not recommended, as it may increase spasticity, which can lead to reduction in range of motion—as well as difficulty with ambulation. However, recently increasing evidence and systemic reviews have shown that strength training can improve muscle strength in children with CP with no adverse effects on spasticity. These results indicate that FPRE training in CP leads to increased muscle power and lower extremity muscle strength in children with CP and could be considered for use in rehabilitation programs.

### 4.2. Structure of Quadriceps in Rehabilitative Ultrasound Imaging

RUSI uses USI to aid rehabilitation of neuromusculoskeletal disorders. Physical therapists have used RUSI to evaluate function and related soft tissue morphology and muscle during exercise and physical tasks [30]. Muscle cross-sectional area has a direct relationship with the capacity of muscle to produce power [7]. RUSI was used to assess thickness of the quadriceps and cross-sectional area of the rectus femoris in order to provide a clinical measurement of increased quadricep volume, which indicates increase of lower extremity strength [44].

In this study, changes in thickness and cross-sectional area of the quadriceps were assessed with RUSI. The time × group interaction effects (*p* < 0.05) were observed for TQ of the dominant and non-dominant sides and CSA of the dominant side. Lee et al. [45] performed progressive functional training on 26 children with CP with spasticity. For 6 weeks, neurodevelopmental treatment and FPRE were performed in the experimental group. The muscle thickness of the quadriceps femoris (QF), cross-sectional area of the rectus femoris (RF) and pennation angle of the gastrocnemius (GCM) were measured with RUSI, and results after the intervention showed significant improvement on those variables (*p* < 0.05). In the experimental group, QF thickness increased from 1.6 cm to 1.9 cm and RF CSA increased from 1.2 cm^2^ to 2.1 cm^2^ [45]. Additionally, increase in structure-related measurements is strongly correlated with increased muscle strength [7].

The outcomes may result from the fact that motor units work in an inadequate, irregular and slower than normal manner following upper motor neuron damage. Therefore, the more affected side cannot activate as normal muscle [17,46]. Thus, it is difficult to generalize this information to all pediatric cases, since this study was not conducted among normally developing children; however, increments observed in the results indicate that FPRE protocol has a positive effect on increasing muscle strength in children with spastic CP.

### 4.3. Muscle Tone According to Popliteal Angle in Passive, Speed and Active Ranges of Motion

Individual spastic muscle fibers with increased tensile strength are stiffer than controls; therefore, to elongate a spastic muscle fiber, more force is needed [47]. Developments of contractures and passive stiffness could be the result of weakened agonist muscles that result in the inability of the spastic antagonist muscle to elongate, thereby perpetuating a pattern of weakness [48]. Popliteal angle was measured in passive, speed and active conditions to determine the impact of FPRE on muscle tone of the hamstring. The FPRE is an exercise program which allows the co-contraction of both quadriceps and hamstring, thereby increasing agonist muscle strength to enable elongation of the antagonist muscle to reduce muscle tone of the lower extremity and increase the popliteal angle.

In this study, popliteal angles in passive, speed and active ranges of motion were assessed in order to evaluate the effects of FPRE on lower leg range of motion and strength. Time × group interaction effects (*p* < 0.05) were observed for the PA-P, PA-S and PA-A of the dominant and non-dominant side. Results of studies by Stubbs, P.W et al. [49] and Scholtes, V.A., et al. [50] imply that muscle strengthening does not increase muscle tone. A prior study has shown that muscle tone was significantly lower after the intervention in the training group (median 1, 0/7; *p* < 0.01) and control group (median 0, 0/4; *p* = 0.02) after implementing a muscle strengthening exercise on children with CP and spasticity, p. A prior study on dynamic strength exercises of the knee extensor showed statistically significant changes (pre: 64.4 to post: 92.6) in the training group and (pre: 60.8 to post: 65.3) control group (*p* < 0.05) [49,50]. Generally, the muscle tension in children with CP develops due to elongated sarcomeres, with decreased action and myosin interaction, which limits the number of cross-bridges causing reduced force production capability [17]. This biomechanical disadvantage may disrupt the ability of the muscle to sufficiently contract to produce the required functional movement.

This study suggests that an increase in passive range of motion allows for adequate muscle length to produce maximum muscle contraction, which could be the reason for an increment in active range of motion. An increase in agonist muscle strength may be strongly correlated with increment in speed range of motion due to co-activation of the thigh muscle. This result provides valid evidence of the effect of FPRE, which can be used in the future to treat children with CP.

### 4.4. Dynamic Balance

A comparison of normally developing children-with-children with CP reveals that children with CP have delayed onset of postural muscle activity. In addition, there is a high level of co-activation of agonist and antagonist muscles at a joint and multiple muscle action sequences are impaired. This can cause difficulty in balance control in CP [4,51]. In this study, dynamic balance was examined using the FRT. An increase in lower extremity muscle strength was shown to be closely correlated with increased dynamic balance ability, which was assessed with the modified FRT [52].

In this study, dynamic balance was assessed with the modified functional reach test in two different positions: forward reaching position and side reaching position. Time effects were observed with the S-FRT (*p* < 0.05), while time × group interaction effects were observed with the F-FRT and S-FRT (*p* < 0.05). FPRE program comprises exercises such as sit to stand and side step-up with load. This exercise program includes voluntary co-contraction of both lower extremity muscles, quadriceps and hamstring muscles [53]. After performing the sit to stand exercise, the FRT value increased in the exercise group; results for the asymmetric paretic limb position in the control group were pre 19.36 ± 6.42 to post 23.43 ± 3.85, while in the training group results were pre 14.91 ± 6.11 to post 21.81 ± 4.59, which indicates that the training group showed statistical improvement after intervention t(*p*); −2.287(0.034) (*p* < 0.05).

In our FPRE study, the FRT was measured in two positions, position forward (F) reaching and side (S) reaching in the FPRE group. These results imply that the exercise protocol of the FPRE, which includes co-activation and functional strengthening, can have a positive effect on dynamic balance.

### 4.5. GMFM in Functional Ability

It is important to accurately measure changes in the acquisition of total motor skills to determine the impact on rehabilitation and the effectiveness of the intervention program in children with CP. The GMFM-88 is an effective measuring tool to detect changes in gross motor function in children with CP [35]. In spastic diplegia CP, strength was highly related to functional abilities [54].

In this study, functional ability was assessed with the GMFM-88. A paired *t*-test revealed a statistically significant increase after the intervention on the GMFM score in the FPRE group (*p* < 0.05).

Ross et al. [54] indicates that lower extremity strength has a strong correlation with functional ability. They conducted a study to determine the relationship between strength and GMFM-66 on CP. The study included 49 boys and 48 girls; mean age ± standard deviation, 9.11 ± 4.8 years. Aggregate strength consisting of values for the ankle dorsiflexors and plantar flexors, knee extensors and flexors and hip abductors and adductors averaged across sides was strongly correlated to the GMFM-66 (r.83). In this study, the GMFM-88 score of the FPRE group increased from a pre-mean value of 69.98 ± 21.55 to a post mean value of 71.78 ± 21.05, (0.019, *p* < 0.05) compared to the control group in which there was a reduction in the GMFM-88 score from 68.15 ± 27.15 to 63.48 ± 27.48 after the intervention. Although an increase was shown in the FPRE group, the change between the two groups was not statistically significant. This result may be due to the duration of the intervention. The results of the study conducted by Bryant et al. [55] indicate that a six-week program can show significant difference on GMFM-88D scores, but not on GMFM-66 or GMFM-88E scores.

The RUSI is an effective assessment device, which can successfully measure thickness and cross-sectional area of the quadriceps, which are associated with lower extremity strength [30]. Quadriceps thickness can also be an indicator of muscle strength, Ohata et al. [8] identified a relationship between thickness of the quadriceps and activity limitation in children and adolescents with CP. Muscle thickness of the quadriceps showed a significant correlation with the GMFM-66 score (*r* = 0.52, *p* = 0.001, 95% CI 0.24 to 0.72). This result suggests that lower extremity muscle thickness may be strongly correlated with functional ability of the child with CP.

This study has the following limitations: a short 6-week intervention period and a small sample size. This makes it difficult to generalize the findings to all children with CP. It is also difficult to control all the factors that may affect the child’s activities of daily living.

## 5. Conclusions

Present study was conducted with twenty-five children to determine the effects of FPRE on strength, lower extremity structure, muscle tone, dynamic balance and functional ability in children with spastic cerebral palsy. This study confirmed that FPRE exerts a positive effect by increasing lower extremity strength and morphology of quadriceps muscle, reducing muscle tone and increasing dynamic balance and functional ability in children with spastic CP. Therefore, we suggest FPRE as an effective, safe and convenient intervention that can be implemented in a six-week period in children with CP in rehabilitation.

## Figures and Tables

**Table 1:** Functional progressive resistance exercise protocol.

FPRE	Exercise Protocol	Duration
Warmup	Range of motion mobilization, stretching	3 min
% weight ^a^	Sit to stand	5 min
	Rest	
% weight ^a^	Half-kneeling standing up, side-step-up	10 min
	Rest	
Body weight	Half-kneeling standing up, side-step-up	10 min
Cooldown	Range of motion mobilization, stretching	2 min

FPRE—functional progressive resistive exercise; ^a^ Progressively increased to five times, 5% weight in 1–2 weeks; 10 times, 10% weight in 3–4 weeks; 15 times, 35% weight in 5–6 weeks.

**Table 2:** General Characteristics of subjects (*N* = 25).

Characteristics	FPRE Group (*n* = 13)	CG Group (*n* = 12)	X^2^/*t*(*p)*
Gender (male/female)	4/9	8/4	1.845(0.078)
Dominant/non-dominant	7/6	9/3	1.082(0.290)
Age (years)	5.54 ± 1.808	7.17 ± 2.167	−2.046(0.052)
Height (cm)	108.54 ± 14.65	117.10 ± 12.73	−1.553(0.134)
Weight (kg)	19.56 ± 7.40	24.37 ± 7.73	−1.587(0.126)
BMI (Z-score)	0.14 ± 1.76	0.60 ± 1.01	−0.790(0.406)
GMFCS level	2.08 ± 0.862	2.33 ± 1.073	−0.661(0.515)
GMFM score	69.98 ± 21.55	68.15 ± 27.15	0.187(0.853)

Values expressed as mean ± standard deviation; FPRE—functional progressive resistive exercise; CG—control group; GMFCS—gross motor function classification system; GMFM—gross motor function measure.

**Table 3:** Knee extensor muscle strength (*N* = 25).

Muscle Strength		FPRE Group (*n* = 13)	CG Group (*n* = 12)	Time	Group	Time × Group
				*F*(*p*)	*F*(*p*)	*F*(*p*)
Non-dominant (*N*)	Pretest	40.62 ± 30.61	34.54 ± 28.55	8.367(0.008)	0.490(0.491)	0.629(0.436)
	Posttest	51.24 ± 33.58	40.59 ± 29.50			
	t(*p*)	−2.196(0.048)	−2.078(0.062)			
Dominant (*N*)	Pretest	30.45 ± 27.57	41.61 ± 34.00	8.368(0.008)	0.060(0.808)	5.412(0.029)
	Posttest	52.39 ± 33.13	43.12 ± 32.17			
	*t*(*p*)	−3.065(0.010)	−0.590(0.567)			

Values expressed as mean ± standard deviation; FPRE—functional progressive resistance exercise; CG—control group.

**Table 4:** Comparison of the structure of the quadriceps between the of functional progressive resistance exercise (FPRE) group and control group (*N* = 25).

Structure			FPRE Group (*n* = 13)	CG Group (*n* = 12)	Time	Group	Time × Group
					*F*(*p*)	*F*(*p*)	*F*(*p*)
TQ	Non-dominant	Pretest	1.39 ± 0.27	1.46 ± 0.29	32.191(0.000)	1.427(0.244)	7.834(0.010)
		Posttest	1.98 ± 0.316	1.66 ± 0.34			
		*t*(*p*)	−8.544(0.000)	−1.610(0.136)			
	Dominant	Pretest	1.41 ± 0.24	1.40 ± 0.308	109.633(0.000)	1.308(0.265)	8.978(0.006)
		Posttest	1.95 ± 0.29	1.70 ± 0.35			
		*t*(*p*)	−11.284(0.000)	−4.578(0.001)			
CSA	Non-dominant	Pretest	3.41 ± 0.807	3.22 ± 0.57	10.288(0.004)	5.240(0.032)	3.830(0.063)
		Posttest	4.54 ± 0.97	3.49 ± 1.05			
		*t*(*p*)	−3.390(0.005)	−0.987(0.345)			
	Dominant	Pretest	3.64 ± 0.64	3.29 ± 0.66	8.578(0.008)	8.549(0.008)	4.451(0.044)
		Posttest	4.63 ± 0.99	3.45 ± 0.89			
		*t*(*p*)	−3.110(0.009)	−0.722(0.485)			

Values expressed as mean ± standard deviation; FPRE—functional progressive resistive exercise; CG—control group; TQ—thickness of the quadriceps; CSA—cross-sectional area of the rectus femoris.

**Table 5:** Muscle tone popliteal angle in passive, speed and active ranges of motion (*N* = 25).

Popliteal Angle			FPRE Group (*n* = 13)	CG Group (*n* = 12)	Time	Group	Time × Group
					*F*(*p*)	*F*(*p*)	*F*(*p*)
PA-P (degree)	Non-dominant	Pretest	151.17 ± 17.82	142.92 ± 18.07	0.241(0.628)	4.464(0.046)	8.994(0.006)
		Posttest	158.57 ± 14.66	137.61 ± 21.35			
		*t*(*p*)	−2.454(0.030)	1.792(0.101)			
	Dominant	Pretest	153.39 ± 15.86	144.68 ± 15.96	4.747(0.040)	6.289(0.020)	4.890(0.037)
		Posttest	163.27 ± 10.19	144.61 ± 16.32			
		*t*(*p*)	−2.745(0.018)	0.028(0.978)			
PA-S (degree)	Non-dominant	Pretest	141.32 ± 13.27	136.49 ± 19.11	0.390(0.538)	5.721(0.025)	8.909(0.007)
		Posttest	152.45 ± 13.11	129.21 ± 20.01			
		*t*(*p*)	−2.879(0.014)	1.495(0.163)			
	Dominant	Pretest	142.24 ± 15.31	140.50 ± 22.41	1.073(0.311)	1.574(0.222)	5.052(0.034)
		Posttest	152.20 ± 14.58	136.82 ± 21.53			
		*t*(*p*)	−2.030(0.065)	1.081(0.303)			
PA-A (degree)	Non-dominant	Pretest	130.96 ± 21.51	128.32 ± 25.78	20.395(0.000)	0.927(0.346)	4.639(0.042)
		Posttest	149.58 ± 19.94	134.91 ± 26.60			
		*t*(*p*)	−4.517(0.001)	−1.774(0.104)			
	Dominant	Pretest	130.48 ± 30.72	126.74 ± 27.70	21.522(0.000)	1.642(0.213)	16.072(0.001)
		Posttest	151.93 ± 20.55	128.30 ± 29.63			
		*t*(*p*)	−4.964(0.000)	−0.722(0.485)			

Values expressed as mean ± standard deviation; FPRE—functional progressive resistive exercise; CG—control group; PA-P—popliteal angle in passive range of motion; PA-S—popliteal angle in speed range of motion; PA-A—popliteal angle in active range of motion.

**Table 6:** Modified functional reach test (forward and side) (*N* = 25).

Balance		FPRE Group (*n* = 13)	CG Group (*n* = 12)	Time	Group	Time × Group
				*F*(*p*)	*F*(*p*)	*F*(*p*)
F-FRT (cm)	Pretest	21.62 ± 6.87	28.17 ± 14.49	0.842(0.368)	0.459(0.505)	10.259(0.004)
	Posttest	26.65 ± 7.92	25.37 ± 10.20			
	*t*(*p*)	−5.635(0.000)	1.186(0.261)			
S-FRT (cm)	Pretest	11.57 ± 5.72	15.52 ± 10.43	6.344(0.019)	0.408(0.529)	4.361(0.048)
	Posttest	16.21 ± 5.37	15.95 ± 8.266			
	*t*(*p*)	−3.734(0.003)	−0.270(0.793)			

Values expressed as mean ± standard deviation; FPRE—functional progressive resistance exercise; CG—control group; F-FRT—forward functional reach test; S-FRT—side functional reach test.

**Table 7:** GMFM score: Pre and post training and changes (*N* = 25).

Gross Motor Function		FPRE Group (*n* = 13)	CG Group (*n* = 12)	Time	Group	Time × Group
				*F*(*p*)	*F*(*p*)	*F*(*p*)
GMFM score	Pretest	69.98 ± 21.55	68.15 ± 27.15	0.346(0.562)	0.288(0.597)	1.744(0.200)
	Posttest	71.78 ± 21.05	63.48 ± 27.48			
	*t*(*p*)	−2.696(0.019)	0.924(0.375)			

Values expressed as mean ± standard deviation; FPRE—functional progressive resistance exercise; CG—control group; GMFM—gross motor function measure.

## References

[1] Richards C.L., Malouin F. (2013). Cerebral palsy: Definition, assessment and rehabilitation. Handb. Clin. Neurol..

[2] Green L.B., Hurvitz E.A. (2007). Cerebral palsy. Phys. Med. Rehabil. Clin. N. Am..

[3] Himmelmann K., Uvebrant P. (2014). The panorama of cerebral palsy in Sweden. XI. Changing patterns in the birth-year period 2003–2006. Acta Paediatr..

[4] Nashner L., Shumway-Cook A., Marin O. (1983). Stance posture control in select groups of children with cerebral palsy: Deficits in sensory organization and muscular coordination. Exp. Brain Res..

[5] Braun K.V.N., Christensen D., Doernberg N., Schieve L., Rice C., Wiggins L., Schendel D., Yeargin-Allsopp M. (2015). Trends in the Prevalence of Autism Spectrum Disorder, Cerebral Palsy, Hearing Loss, Intellectual Disability, and Vision Impairment, Metropolitan Atlanta, 1991–2010. PLoS ONE.

[6] Lampe R., Grassl S., Mitternacht J., Gerdesmeyer L., Gradinger R. (2006). MRT-measurements of muscle volumes of the lower extremities of youths with spastic hemiplegia caused by cerebral palsy. Brain Dev..

[7] Moreau N.G., Simpson K.N., Teefey S.A., Damiano D.L. (2010). Muscle Architecture Predicts Maximum Strength and Is Related to Activity Levels in Cerebral Palsy. Phys. Ther..

[8] Ohata K., Tsuboyama T., Haruta T., Ichihashi N., Kato T., Nakamura T. (2008). Relation between muscle thickness, spasticity, and activity limitations in children and adolescents with cerebral palsy. Dev. Med. Child Neurol..

[9] Aviram R., Harries N., Namourah I., Amro A., Bar-Haim S. (2016). Effects of a group circuit progressive resistance training program compared with a treadmill training program for adolescents with cerebral palsy. Dev. Neurorehabilit..

[10] Hoffman R., Corr B.B., Stuberg W.A., Arpin D., Kurz M.J. (2018). Changes in lower extremity strength may be related to the walking speed improvements in children with cerebral palsy after gait training. Res. Dev. Disabil..

[11] Fowler E.G., Kolobe T.H., Damiano D.L., Thorpe D.E., Morgan D.W., Brunstrom J.E., Coster W.J., Henderson R.C., Pitetti K.H., Rimmer J.H. (2007). Promotion of Physical Fitness and Prevention of Secondary Conditions for Children With Cerebral Palsy: Section on Pediatrics Research Summit Proceedings. Phys. Ther..

[12] Miller T.L. (2007). A hospital-based exercise program to improve body composition, strength, and abdominal adiposity in 2 HIV-infected children. AIDS Read..

[13] Kelly M., Darrah J. (2005). Aquatic exercise for children with cerebral palsy. Dev. Med. Child Neurol..

[14] Dunst C.J. (2017). Research Foundations for Evidence-Informed Early Childhood Intervention Performance Checklists. Educ. Sci..

[15] Cameron R.A., Brousseau J., Cerracchio K., Clark T., Cotsalas T., Eisen K., Gannotti M.E. (2018). Multimodal Community-Based Exercise for Children with Cerebral Palsy: Dosing Interventions for Effectiveness. Crit. Rev. Phys. Rehabilit. Med..

[16] Ozal C., Türker D., Korkem D. (2016). Strength Training in People with Cerebral Palsy. Cereb. Palsy Curr. Steps.

[17] Mockford M., Caulton J.M. (2010). The Pathophysiological Basis of Weakness in Children With Cerebral Palsy. Pediatr. Phys. Ther..

[18] Clutterbuck G.L., Auld M., Johnston L. (2018). Active exercise interventions improve gross motor function of ambulant/semi-ambulant children with cerebral palsy: A systematic review. Disabil. Rehabilit..

[19] Faigenbaum A.D., Kraemer W.J., Blimkie C.J.R., Jeffreys I., Micheli L.J., Nitka M., Rowland T.W. (2009). Youth Resistance Training: Updated Position Statement Paper From the National Strength and Conditioning Association. J. Strength Cond. Res..

[20] Damiano D.L. (2014). Progressive resistance exercise increases strength but does not improve objective measures of mobility in young people with cerebral palsy. J. Physiother..

[21] Dos Santos A.N., Pavão S.L., Rocha N. (2011). Sit-to-stand movement in children with cerebral palsy: A critical review. Res. Dev. Disabil..

[22] Mukherjee A., Chakravarty A. (2010). Spasticity Mechanisms—For the Clinician. Front. Neurol..

[23] Palisano R., Rosenbaum P., Walter S., Russell D., Wood E., Galuppi B. (1997). Development and reliability of a system to classify gross motor function in children with cerebral palsy. Dev. Med. Child Neurol..

[24] Scholtes V.A., Becher J.G., Janssen-Potten Y.J., Dekkers H., Smallenbroek L., Dallmeijer A.J. (2012). Effectiveness of functional progressive resistance exercise training on walking ability in children with cerebral palsy: A randomized controlled trial. Res. Dev. Disabil..

[25] Dahab K.S., McCambridge T.M. (2009). Strength Training in Children and Adolescents: Raising the Bar for Young Athletes?. Sports Health.

[26] Verschuren O., Ada L., Maltais D.B., Gorter J.W., Scianni A., Ketelaar M. (2011). Muscle Strengthening in Children and Adolescents With Spastic Cerebral Palsy: Considerations for Future Resistance Training Protocols. Phys. Ther..

[27] Winter D.A., Wells R.P., Orr G.W. (1981). Errors in the use of isokinetic dynamometers. Eur. J. Appl. Physiol. Occup. Physiol..

[28] Chesterton L.S., Sim J., Wright C.C., Foster N.E. (2007). Interrater Reliability of Algometry in Measuring Pressure Pain Thresholds in Healthy Humans, Using Multiple Raters. Clin. J. Pain.

[29] Teyhen D., Koppenhaver S. (2011). Rehabilitative ultrasound imaging. J. Physiother..

[30] Fernández-Carnero J., Arias-Buría J.L., Zaldívar J.N.C., Quiñones A.L., Calvo-Lobo C., Martín-Saborido C. (2019). Rehabilitative Ultrasound Imaging Evaluation in Physiotherapy: Piloting a Systematic Review. Appl. Sci..

[31] Sarikaya I.A., Inan M., Seker A. (2015). Improvement of popliteal angle with semitendinosus or gastrocnemius tenotomies in children with cerebral palsy. Acta Orthop. Traumatol. Turc..

[32] Domínguez G., Cardiel E., Arias S., Rogeli P. A Digital Goniometer Based on Encoders for Measuring Knee-Joint Position in An Orthosis. Proceedings of the 2013 World Congress on Nature and Biologically Inspired Computing.

[33] Duncan P.W., Weiner D.K., Chandler J., Studenski S. (1990). Functional Reach: A New Clinical Measure of Balance. J. Gerontol..

[34] Mason A.N., Lum J., Milligan A., Robinson J. (2018). Functional Reach Test. Crit. Rev. Phys. Rehabilit. Med..

[35] Alotaibi M., Long T., Kennedy E., Bavishi S. (2013). The efficacy of GMFM-88 and GMFM-66 to detect changes in gross motor function in children with cerebral palsy (CP): A literature review. Disabil. Rehabilit..

[36] Lee J.H., Lim H.K., Park E., Song J., Lee H.S., Ko J., Kim M. (2013). Reliability and Applicability of the Bayley Scale of Infant Development-II for Children With Cerebral Palsy. Ann. Rehabilit. Med..

[37] Orlin M.N., Palisano R.J., Chiarello L.A., Kang L.-J., Polansky M., Almasri N., Maggs J. (2009). Participation in home, extracurricular, and community activities among children and young people with cerebral palsy. Dev. Med. Child Neurol..

[38] Wiley M.E., Damiano D.L. (1998). Lower-extremity strength profiles in spastic cerebral palsy. Dev. Med. Child Neurol..

[39] Lee J.H., Sung I.Y., Yoo J.Y. (2008). Therapeutic effects of strengthening exercise on gait function of cerebral palsy. Disabil. Rehabilit..

[40] Liao H.-F., Liu Y.-C., Liu W.-Y., Lin Y.-T. (2007). Effectiveness of Loaded Sit-to-Stand Resistance Exercise for Children With Mild Spastic Diplegia: A Randomized Clinical Trial. Arch. Phys. Med. Rehabilit..

[41] McBurney H., Taylor N.F., Dodd K.J., Graham H.K. (2003). A qualitative analysis of the benefits of strength training for young people with cerebral palsy. Dev. Med. Child Neurol..

[42] Dodd K.J., Taylor N.F., Damiano D.L. (2002). A systematic review of the effectiveness of strength-training programs for people with cerebral palsy. Arch. Phys. Med. Rehabilit..

[43] Anttila H., Autti-Rämö I., Suoranta J., Mäkelä M., Malmivaara A. (2008). Effectiveness of physical therapy interventions for children with cerebral palsy: A systematic review. BMC Pediatr..

[44] Ramírez-Fuentes C., Mínguez-Blasco P., Ostiz F., Sánchez-Rodríguez D., Messaggi-Sartor M., Macías R., Muniesa J.M., Rodríguez D.A., Vila J., Perkisas S. (2018). Ultrasound assessment of rectus femoris muscle in rehabilitation patients with chronic obstructive pulmonary disease screened for sarcopenia: Correlation of muscle size with quadriceps strength and fat-free mass. Eur. Geriatr. Med..

[45] Lee M., Ko Y., Shin M.M.S., Lee W. (2015). The effects of progressive functional training on lower limb muscle architecture and motor function in children with spastic cerebral palsy. J. Phys. Ther. Sci..

[46] Rose J., McGill K.C. (2005). Neuromuscular activation and motor-unit firing characteristics in cerebral palsy. Dev. Med. Child Neurol..

[47] Fridén J., Lieber R.L. (2003). Spastic muscle cells are shorter and stiffer than normal cells. Muscle Nerve.

[48] Toner L.V., Cook K., Elder G.C.B. (1998). Improved ankle function in children with cerebral palsy after computer-assisted motor learning. Dev. Med. Child Neurol..

[49] Stubbs P.W., Diong J. (2016). The effect of strengthening interventions on strength and physical performance in people with cerebral palsy (PEDro synthesis). Br. J. Sports Med..

[50] Scholtes V.A., Dallmeijer A.J., Rameckers E., Verschuren O., Tempelaars E., Hensen M., Becher J.G. (2008). Lower limb strength training in children with cerebral palsy—A randomized controlled trial protocol for functional strength training based on progressive resistance exercise principles. BMC Pediatr..

[51] Burtner P., Qualls C., Woollacott M. (1998). Muscle activation characteristics of stance balance control in children with spastic cerebral palsy. Gait Posture.

[52] Saquetto M., Carvalho V., Silva C., Conceição C., Gomes-Neto M. (2015). The effects of whole body vibration on mobility and balance in children with cerebral palsy: A systematic review with meta-analysis. J. Musculoskelet. Neuronal Interact..

[53] Ross S.M., Macdonald M., Bigouette J.P. (2016). Effects of strength training on mobility in adults with cerebral palsy: A systematic review. Disabil. Health J..

[54] Ross S.A., Engsberg J.R. (2007). Relationships between Spasticity, Strength, Gait, and the GMFM-66 in Persons With Spastic Diplegia Cerebral Palsy. Arch. Phys. Med. Rehabilit..

[55] Bryant E.C., Pountney T., Williams H., Edelman N. (2012). Can a six-week exercise intervention improve gross motor function for non-ambulant children with cerebral palsy? A pilot randomized controlled trial. Clin. Rehabilit..

